# Serum Globulin to Albumin Ratio as a Novel Predictor of Adverse Clinical Outcomes in Coronary Artery Disease Patients Who Underwent PCI

**DOI:** 10.31083/j.rcm2410278

**Published:** 2023-10-07

**Authors:** Si-Fan Wang, Ting-Ting Wu, Ying-Ying Zheng, Xian-Geng Hou, Hai-Tao Yang, Yi Yang, Xiang Xie

**Affiliations:** ^1^Department of Cardiology, First Affiliated Hospital of Xinjiang Medical University, 830054 Urumqi, Xinjiang, China

**Keywords:** albumin, serum globulin to albumin ratio, coronary artery disease, all-cause death long-term prognosis

## Abstract

**Background:**

Coronary heart disease is one of the main causes of Mortality. Many biological indicators have been used to predict the prognosis of patients with coronary heart disease. The ratio of serum globulin to albumin (GAR) has been used to predict the prognosis of patients with various cancers. It has been proven that GAR is related to the prognosis of patients with stroke. However, GAR’s role in cardiovascular disease remains unclear. Our purpose was to investigate the predictive value of GAR on clinical outcomes in post-percutaneous coronary intervention (PCI) patients with coronary artery disease (CAD).

**Methods:**

From Dec. 2016 to Oct. 2021, a total of 14,994 patients undergoing PCI patients admitted to the First Affiliated Hospital of Xinjiang Medical University were divided into high GAR group (GAR ≥0.76, n = 4087) and low GAR group (GAR <0.76, n = 10,907). The incidence of adverse outcomes including all-cause mortality (ACM), cardiovascular mortality (CM), major adverse cardiovascular events (MACE) and major adverse cardiovascular and cerebrovascular events (MACCE) was compared between the two groups. Multivariate Cox regression was used to adjust for the effects of confounding factors, while hazard ratios (HRs) and 95% confidence intervals (95% CI) were calculated. Median follow-up time was 24 months.

**Results:**

Compared with the low GAR group, the high GAR group had significantly higher incidence of ACM (6.5% vs. 1.7%, *p* < 0.001); CM (4.9% vs. 1.2%, *p* < 0.001), MACE (10.5% vs. 6.7%, *p* < 0.001), and MACCE (11.3% vs. 7.5%, *p* < 0.001). Cox regression analysis showed the patients in the high GAR group had a 1.62-fold increased risk for ACM (HR = 2.622, 95% CI: 2.130–3.228, *p* < 0.01), a 1.782-fold increased risk for CM (HR = 2.782, 95% CI: 2.180–3.550, *p* < 0.01). There was a 37.2% increased risk for MACE (HR = 1.372, 95% CI: 1.204–1.564, *p* < 0.01), and 32.4% increased risk for MACCE (HR = 1.324, 95% CI: 1.169–1.500,* p* < 0.01), compared to the patients in the low GAR group.

**Conclusions:**

The present study suggested that post-PCI CAD patients with higher GAR presented significantly increased mortality and adverse events GAR level at admission may 296 be considered as part of risk stratification when PCI is possible in patients with coronary heart disease.

**Clinical Trial Registration:**

The detailed information of the PRACTICE study has been registered on http://Clinicaltrials.gov (Identifier: NCT05174143).

## 1. Introduction

The number of people in China who suffer from cardiovascular disease has been growing as a result of an aging society and a high prevalence of unhealthy lifestyles. Coronary artery disease (CAD) has become one of the diseases that seriously threaten human health [1], and caused 365,914 deaths worldwide in 2017. About two out of every 10 deaths from coronary heart disease occur in adults under 65. In developed countries, millions of patients with chest pain are typically hospitalized each year. About 50% of them were diagnosed with coronary heart disease, including stable angina pectoris, unstable angina pectoris and acute myocardial infarction [2]. That causes huge economic burden to patients and society. It is reported that at the beginning of 2010, the total hospitalization cost of cardiovascular disease patients exceeded ¥40 billion, accounting for more than 1.60% of the national health expenditure [3]. With the percutaneous coronary intervention (PCI) technology’s ongoing advancements, PCI has revolutionized the management of CAD patients [4]. However, adverse clinical outcomes continue to occur in some patients treated with PCI [5]. Current predictors for assessing the prognosis of CAD have included inflammatory marker, low density lipoprotein cholesterol (LDL-C), hypertension, diabetes, smoking, and other relevant factors affecting cardiovascular disease [6]. The clinical value of serum albumin (ALB) and other functional proteins for the prognostic assessment of CAD has been well documented [7]. Serum ALB, the main protein contained in human plasma, not only has anti-inflammatory and anti-platelet aggregation effects but also is the most significant antioxidant in whole blood [8]. Low levels of serum ALB have now become an independent predictor of many cardiovascular diseases. It has been shown that low serum ALB levels are significantly associated with the occurrence of MACE [9]. A meta-analysis [10] proved that low levels of serum ALB were associated with an increased risk of CAD. Moreover, a study by Zhu L *et al*. [11] found that low levels of serum ALB were a strong predictor of all-cause mortality in patients with the acute coronary syndrome (ACS). Among the globulins (GLB), immunoglobulins are their main components, which partly reflect the inflammatory condition of the body [12]. Immune inflammation plays an important role in the occurrence of atherosclerosis. The formation, growth, differentiation and rupture of atherosclerotic plaques are all affected by the immune system [13]. The globulin-to-albumin ratio (gar) is the ratio of the serum globulin level and the serum albumin level, it reflects the systemic inflammatory response and has been used to predict the poor prognosis of various cancers and chronic diseases in previous reports [14]. Studies by Takayuki Shimizu [15] have shown that GAR can predict the prognosis of patients with gastric cancer after radical resection. Hiroyuki Hachiya’s study [16] demonstrated that GAR can be used as an independent predictor of postoperative survival in patients with colon cancer. Chunjian Li’s study [17] demonstrated GAR’s predictive value for 3-month functional prognosis in patients with acute ischemic stroke. However, although GAR has been shown to have predictive value in the prognosis of tumor patients and patients with acute cerebrovascular disease, there are few studies on the relationship between GAR and the prognosis of cardiovascular disease, the relationship between GAR and post-PCI clinical outcomes for CAD patients remains unclear. In our study, we utilized data from a large prospective cohort to analyze the predictive value of GAR for adverse outcomes in post-PCI patients with CAD.

## 2. Subjects and Methods

### 2.1 Subjects

All the patients derived from a single-center prospective cohort study named PRACTICE which was conducted in the First Affiliated Hospital of Xinjiang Medical University from Dec. 2016 to Oct. 2021. The detailed information of the PRACTICE study has been registered on http://Clinicaltrials.gov (Identifier: NCT05174143). In the PRACTICE study, we enrolled 15,250 CAD patients who underwent PCI. Baseline data, including sex, age, smoking, chronic disease history, biochemical data, echocardiographic information, and medication were collected.

Inclusion criteria: (1) At least one of the three main arteries stenosis ≥70%, according to percutaneous coronary artery angiography findings. (2) All patients had received PCI and at least 1 stent was implanted.

Exclusion criteria: (1) Clinical evidence of acute infection, active cancer, hematologic system proliferative disease. (2) Combination of severe congenital heart disease and severe valvular heart disease. (3) Combination of severe hepatic or renal insufficiency 256 patients were excluded according to above exclusion criteria. Finally, 14,994 patients were included in the present study. The study protocol was approved by the ethics committee of the First Affiliated Hospital of Xinjiang Medical University. All patients signed an informed consent form.

### 2.2 Grouping

The area under the curve (AUC) value of GAR for predicting all-cause mortality was calculated as 0.694 using receiver operating characteristic (ROC) curve analysis, and the optimal cut-off value of GAR value was obtained as 0.76. Based on the optimal cut-off value, patients were divided into the low-value GAR group (GAR <0.76, n = 10,907 cases) and the high-value GAR group (GAR ≥0.76, n = 4087 cases).

### 2.3 Endpoints and Follow-Ups

In this study, patients were followed up for up to 5 years after discharge by telephone, visit records, and outpatient medical records, with a median follow-up time of 24 months. The primary endpoints at follow-up were mortality, including all-cause and cardiovascular mortality. Secondary endpoints include major adverse cardiovascular events (MACE), and major adverse cardiac and cerebrovascular events (MACCE). MACE was defined as the composite of cardiovascular death, nonfatal MI, and target vessel revascularization (TVR). MACCE was defined as the composite of MACE and stroke.

### 2.4 Statistical Analyses

Statistical analysis was completed using SPSS 22.0.1 Statistics software (SPSS Inc, Chicago, IL, USA) and R (version 4.1.0, R Foundation for Statistical Computing, Vienna, Austria). The measurement data were expressed as mean ± standard deviation; the normality test was performed before the analysis, and the data conforming to the normal distribution were compared between groups using the Student’s *t*-test (*t*-test); the count data were expressed in the form of cases or rates, and the chi-squared test was used for comparison between the two groups. The multivariable Cox proportional hazards regression analysis was used for multivariable analysis, and the hazard ratio (HR) and its 95% confidence interval (95% CI) were calculated. We performed the Kaplan-Meier survival function to construct survival curves, and the level of statistical significance was set at *p* < 0.05. The ROC curves were drawn based on the ALB, GLB and GAR values collected at admission, and the area under the curve (AUC) was calculated and compared.

## 3. Results

### 3.1 Comparison of Baseline Characteristics of the Two Groups

A comparison of baseline data revealed that the total number of patients in the low GAR group was 10,907, accounting for 72.74% of the total number of people, of whom 8299 (76.1%) were males. The total number of patients in the high GAR group was 4087, accounting for 27.26% of the total number of people, of whom 2783 (68.1%) were males. There were no significant differences between the two groups in blood urea nitrogen, uric acid (UA), and total cholesterol (TC). Significant differences were found in age, smoking, alcohol drinking, family history of CAD, diabetes, hypertension, high density lipoprotein cholesterol (HDL-C), and LDL-C was observed between the two groups (all *p* < 0.05; Table 1).

**Table 1.** **Comparison of baseline characteristics between the two groups**.

Variables	Low GAR group (n = 10,907)	High GAR group (4087)	$t/x^{2}$	*p* values
Age (years)	58.978 ± 11.28	63.28 ± 11.64	–20.301	<0.001
Sex, Male, n (%)	8299 (76.1)	2783 (68.1)	98.543	<0.001
Smoking, n (%)	4515 (41.4)	1421 (34.8)	54.587	<0.001
Drinking, n (%)	2712 (24.9)	831 (20.3)	33.837	<0.001
Family history, n (%)	1303 (12.7)	430 (11.2)	5.395	0.02
Diabetes, n (%)	4628 (42.4)	2442 (59.8)	357.855	<0.001
Hypertension, n (%)	7377 (67.8)	2890 (70.9)	12.643	<0.001
BUN (mmol/L)	8.778 ± 28.760	9.424 ± 26.28	–1.253	0.21
UA (µmol/L)	433.978 ± 576.573	430.191 ± 495.506	0.371	0.71
TCHO (mmol/L)	3.878 ± 1.080	3.886 ± 1.123	–0.362	0.71
HDL-C (mmol/L)	1.070 ± 0.298	1.034 ± 0.321	5.981	<0.001
LDL-C (mmol/L)	2.454 ± 0.889	2.513 ± 0.889	–3.513	<0.001
GLB (g/L)	25.269 ± 3.578	33.483 ± 4.494	–104.756	<0.001
ALB (g/L)	42.731 ± 6.171	36.670 ± 4.208	68.510	<0.001
GAR	0.590 ± 0.136	0.9343 ± 0.398	–54.104	<0.001

Note: BUN, blood urea nitrogen; UA, uric acid; TCHO, total cholesterol; HDL-C, high density lipoprotein cholesterol; LDL-C, low density lipoprotein cholesterol; GLB, globulin; ALB, albumin; GAR, globulin to albumin ratio.

### 3.2 Incidence of Mortality and Adverse Clinical Events

As shown in Table 2, the incidence of ACM (6.5% vs. 1.7%, *p* < 0.001), CM (4.9% vs. 1.2%, *p* < 0.001), MACE (10.5% vs. 6.7%, *p* < 0.001), and MACCE (11.3% vs. 7.5%, *p* < 0.001) in the high GAR group was more frequently compared to that in the low GAR group.

**Table 2.** **Comparison of endpoint events between the two groups**.

Outcomes	Low GAR group (n = 10,907)	High GAR group (n = 4087)	$x^{2}$	*p* values
ACM, n (%)	182 (1.7)	266 (6.5)	240.247	<0.001
CM, n (%)	133 (1.2)	200 (4.9)	184.815	<0.001
MACE, n (%)	731 (6.7)	431 (10.5)	61.432	<0.001
MACCE, n (%)	816 (7.5)	460 (11.3)	54.380	<0.001

Note: ACM, all-cause mortality; CM, cardiovascular mortality; MACE, major adverse cardiovascular events; MACCE, major adverse cardiovascular and cerebrovascular events; GAR, Globulin to albumin ratio.

### 3.3 Kaplan-Meier Survival Analysis

**Fig. 1.**
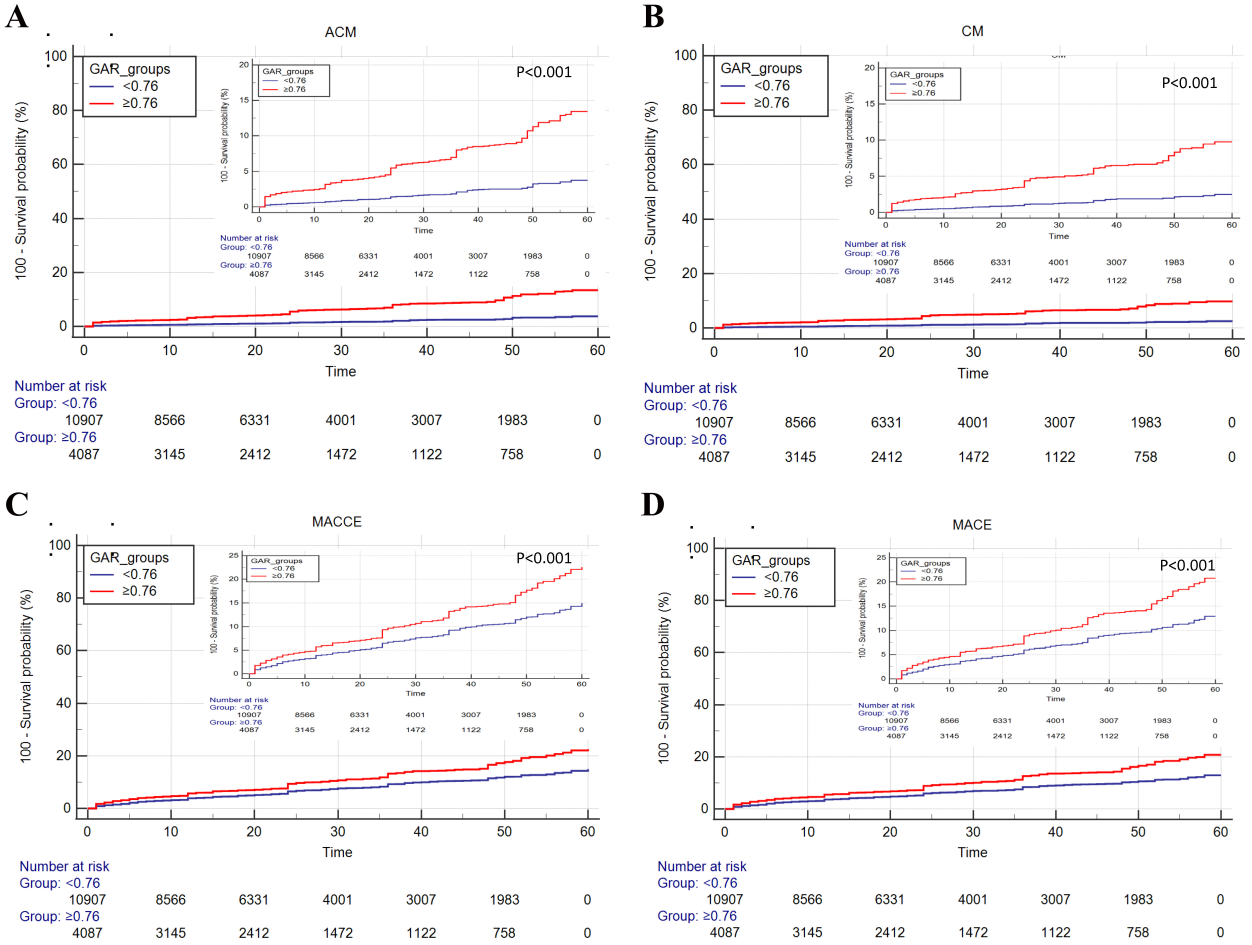
**Cumulative Kaplan-Meier estimates of the time to the first adjudicated occurrence of primary endpoint and secondary endpoints: (A) ACM; (B) CM; (C) MACCE; (D) MACE. **ACM, all-cause mortality; CM, cardiovascular mortality; MACE, major adverse cardiovascular events; MACCE, major adverse cardiovascular and cerebrovascular events; GAR, Globulin to albumin ratio.

Kaplan-Meier survival analysis suggested that the high-GAR group had increased cumulative risk of ACM, CM, MACCE, and MACE (Fig. 1) than those in the low-GAR group.

### 3.4 Multivariate COX Regression Analysis

**Fig. 2.**
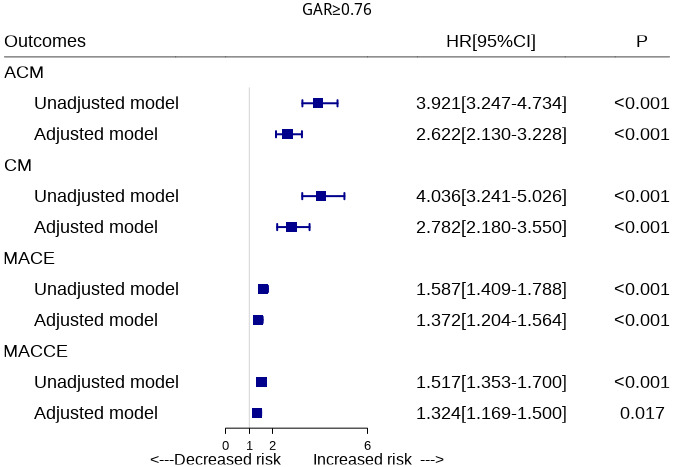
**Unadjusted and adjusted models of association of GAR with outcomes using Cox regression analyses. **ACM, all-cause mortality; CM, cardiovascular mortality; MACE, major adverse cardiovascular events; MACCE, major adverse cardiovascular and cerebrovascular events; GAR, Globulin to albumin ratio; HR, hazard ratio.

After adjusting for confounding factors such as sex, age, smoking, alcohol drinking, BUN, TC, LDL-C, HDL-C, and UA, multivariate Cox regression analysis showed that the risk of ACM increased 1.622-fold (HR = 2.622, 95% CI: 2.130–3.228, *p* < 0.01) in the high GAR group compared with low GAR group. There was a 1.782-fold increase in cardiovascular mortality (HR = 2.782, 95% CI: 2.180–3.550, *p* < 0.01), a 37.2% increase in the risk of MACE events (HR = 1.372, 95% CI: 1.204–1.564, *p* < 0.01), a 32.4% increase in the risk of MACCE events (HR = 1.324, 95% CI: 1.169–1.500, *p* < 0.01, Fig. 2).

### 3.5 Subgroup Analysis

**Fig. 3.**
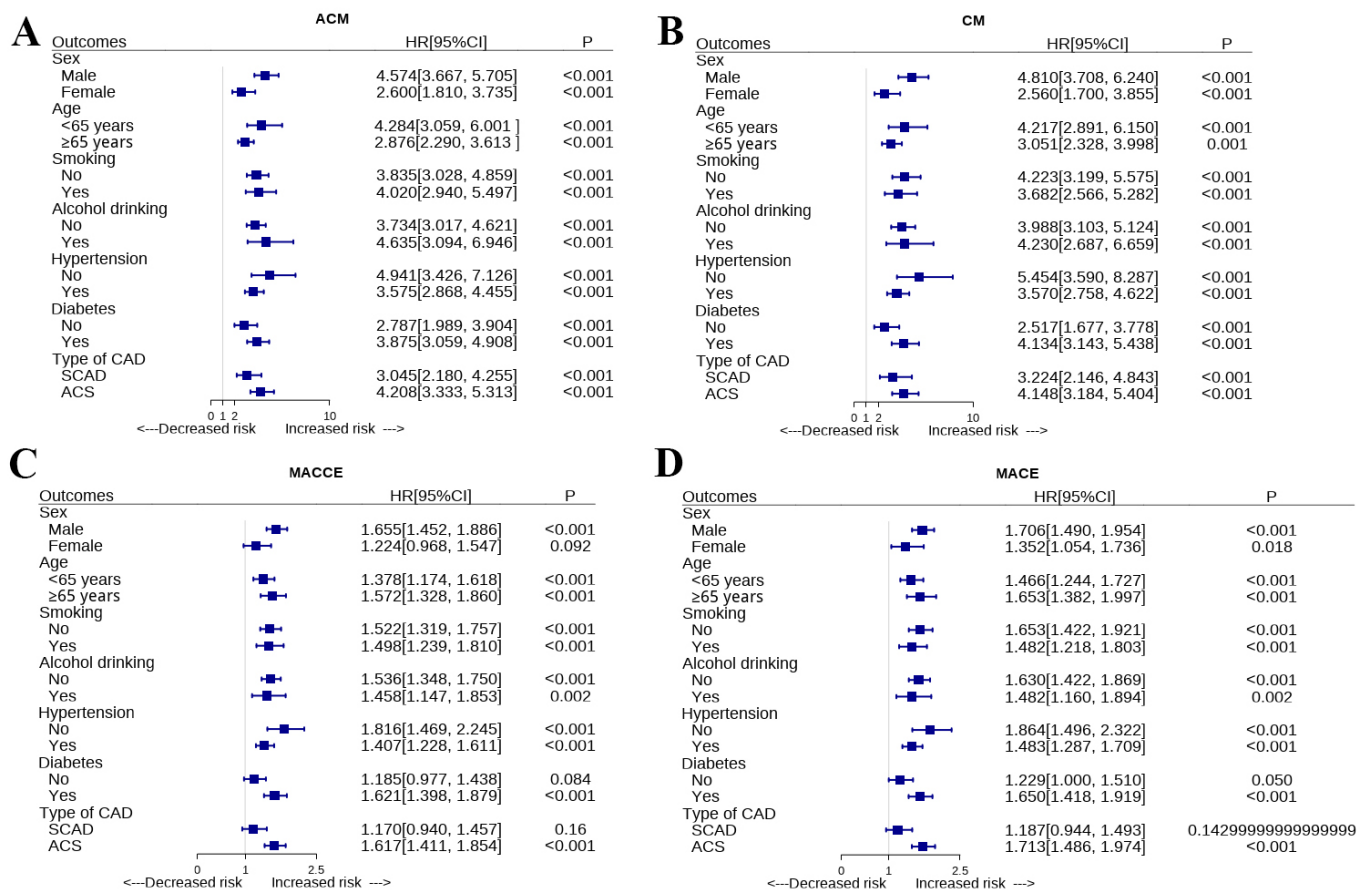
**Subgroups analyses of the relationship between GAR and ACM (A), CM (B), MACE(C) and MACCE (D) according to Age, sex, smoking, alcohol drinking, hypertension, diabetes, and type of CAD. **ACM, all-cause mortality; CM, cardiovascular mortality; MACE, major adverse cardiovascular events; MACCE, major adverse cardiovascular and cerebrovascular events; GAR, Globulin to albumin ratio; CAD, coronary artery disease; HR, hazard ratio; ACS, acute coronary syndrome; SCAD, stable coronary artery disease.

We stratified the overall patients by age, sex, smoking, alcohol drinking, hypertension, diabetes and type of CAD. As shown in Fig. 3, we did not find any influence of age, sex, smoking, alcohol drinking, hypertension, diabetes and type of CAD on the association of GAR with mortality (ACM or CM). However, for MACE or MACCE, we found the association of GAR was modified by sex, diabetes and type of CAD. In the subgroup of female, non-diabetic patients or stable CAD patients, we did not find significant association of GAR with MACE or MACCE.

### 3.6 Comparison of GAR with ALB and GLB Predictive Values

**Fig. 4.**
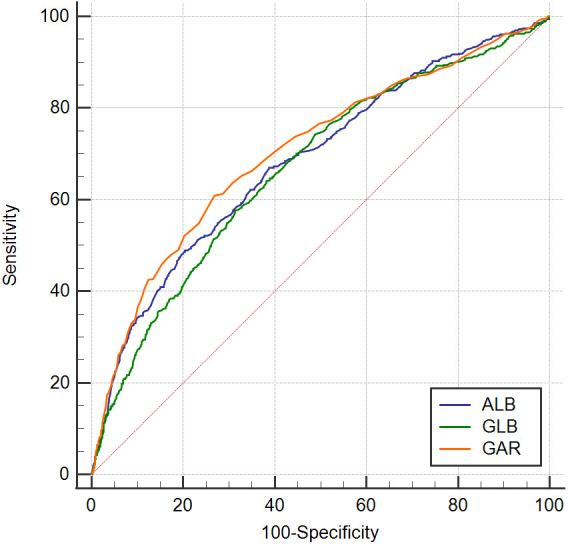
**ROC curve for the ALB, GLB, and GAR for predicting 5‑year mortality.** ROC, receiver operating characteristic; ALB, albumin; GLB, globulins; GAR, Globulin to albumin ratio.

ROC curves were plotted based on ALB, GLB and GAR values collected at patient admission (Fig. 4), and AUC for the occurrence of all-cause mortality was calculated: ALB (AUC = 0.685, 95% CI: 0.678–0.693, *p* < 0.05), GLB (AUC = 0.669, 95% CI: 0.661–0.677, *p* < 0.05), GAR (AUC = 0.706, 95% CI: 0.699–0.713, *p* < 0.05), which demonstrates that GAR is a better predictor of all-cause mortality than ALB and GLB.

## 4. Discussion

The results of the study show that a higher GAR is a significant predictive factor of adverse events in patients undergoing PCI for coronary heart disease. The optimal cut off value of GAR is 0.76, was studied by drawing ROC curve. And the patients were divided into high-value GAR groups and low-value GAR groups according to this value. The high-value groups and the low-value group were independently related to ACM, CM, MACE, and MACCE events. Kaplan–Meier survival analysis suggested that patients with high GAR group exhibited increased accumulated risk of ACM, CM, MACE and MACCE events. The results showed that GAR has an advantage over ALB and GLB in predicting all-cause mortality according to the comparison of areas under the ROC curve.

Cardiovascular disease (CVD) is the leading cause of death in the Chinese population [18]. According to the relevant reports, the prevalence of CVD in China has been still increasing steadily, and the number of CVD patients in China is currently about 330 million, while in 2019, CVD occupied first place in the composition of disease-related death among urban and rural people in China, of which the mortality rate of CAD-related death has reached 121.59/100,000 [19]. In the pathogenesis of CAD, the “theory of endothelial injury - response”, which has been recognized by most researchers, proposes that endothelial cell dysfunction can secrete inflammatory substances that promote leukocyte adherence, adhesion, aggregation, and migration to the subendothelium, thus promoting the development of atherosclerosis (AS) [20]. At the same time, the inflammatory response decreases the stability of the AS plaque leading to its rupture, thus causing ACS [21].

Currently, a variety of inflammatory biomarkers have been found to be associated with coronary heart disease and used as independent predictors to predict the long-term prognosis of patients with coronary heart disease. In recent years, a new inflammatory marker, lymphocyte to monocyte ratio, has been proven in Qian Wang’s study [22] to have a strong independent predictive value for hospitalization and long-term adverse events in patients with ST-segment elevation myocardial infarction after initial PCI. In a meta-study, the neutrophil to lymphocyte ratio (NLR) was demonstrated to be useful for assessing the risk level of patients with St-segment elevation myocardial infarction after PCI [23]. Tomasz Urbanowicz’s research [24] has proven that increased postoperative NLR, as a marker of inflammatory response, is associated with medium-term mortality in Off-pump coronary artery bypass grafting (OPCAB) patients. Therefore, inflammatory markers have important predictive value for the occurrence of adverse events in patients with coronary heart disease after PCI.

GAR is a novel inflammatory marker. We hypothesized that GAR is associated the risk of adverse clinical outcomes mainly through antioxidant and anti-inflammatory responses, and in higher GAR patients the antioxidant and anti-inflammatory response abilities are decreased, thus increasing the risk of adverse clinical outcomes. Serum ALB is synthesized in the liver, which has a variety of physiological functions, including the regulation of coagulation, anti-inflammatory, antioxidant, and maintenance of normal vascular permeability [25]. Serum ALB can bind and transport inflammatory substances and inflammatory mediators thereby regulating systemic and organ inflammatory responses and relieving oxidative stress [26]. Chronic inflammation and infection are associated with coronary heart disease and atherosclerosis. In the inflammatory state, the increased activity of macrophages and other immune system cells leads to the production of cytokines that shift protein synthesis in the liver from serum albumin to other acute phase proteins, resulting in reduced levels [27]. LDL oxidation is one of the early steps in the atherogenic process. Serum ABL inhibits the production of free hydroxyl radicals in the copper-containing ions and $H_{2}$$O_{2}$ system, and can scavenge peroxygen radicals, as well as inhibit the copper-dependent lipid peroxidation system [28]. A growing number of studies have shown that lower serum ALB levels are an independent risk factor for CAD. A study by Meng H *et al*. [29] proved that low serum ALB levels were associated with cardiovascular mortality and incidence. The Prenner SB’s study [30] showed that serum ALB is a strong prognostic factor for heart failure with lower ejection fraction. Suzuki S *et al*. [31] found a predictive effect of low serum ALB levels on the occurrence of MACE events in patients with stable CAD. Hong SI *et al*. [32] found that lower serum ALB levels were also independently associated with death caused by sudden cardiac arrest. According to Wallentin L *et al*. [33], higher GLB is also a risk factor for the development of cardiovascular mortality in patients with CAD. Chenglong Zhang *et al*. [34] demonstrated that globulin was independently correlated with Gensini score and the incidence of three-vessel lesions in patients with ACS, suggesting that globulin may be a qualified indicator for evaluating the severity of coronary artery stenosis, Cheung CL demonstrated that GLB was a predictor of all-cause mortality, cardiovascular mortality, and co-cardiovascular events in multiple groups of patients [35].

The diagnostic biomarker used in this study, the GAR, reflects the ratio between non-ALB proteins and serum ALB. Although most current studies have shown the predictive value of serum ALB for the occurrence of adverse events in patients with CAD, serum ALB concentrations are affected by physiological and pathological conditions in patients when performing laboratory tests, while these conditions have a smaller effect on the GAR. The GAR is a combined indicator of serum ALB and non-ALB proteins, whose predictive significance for CAD is not solely influenced by lower serum ALB or higher GLB. In our study, we compared the ROC curve area of the GAR, GLB, and ALB, for predicting all-cause mortality in patients with CAD, and the results showed that the GAR is more predictive than GLB and ALB alone. Therefore, compared with low serum ALB levels or high GLB levels, the predictive value of the GAR is more advantageous for the occurrence of adverse events in post-PCI patients with CAD.

## 5. Limitations

First of all, this study is a single-center, observational and prospective cohort study, and GAR is a relatively new biological indicator, and its relationship with prognosis still needs to be confirmed by further large-scale studies. Secondly, details about coronary anatomy were not registered. So it is not clear whether GAR’s assessment of long-term prognosis differs among patients according to coronary heart disease severity and PCI outcome.

## 6. Conclusions

This study demonstrates that high GAR can be an independent predictor of adverse events in post-PCI patients with CAD. Moreover, because the ratio of serum globulin to albumin is easy to measure and relatively low in cost, GAR level at admission may be considered as part of risk stratification when PCI is possible in patients with coronary heart disease. In addition, the combination of hypertension, diabetes and other traditional risk factors helps to identify patients with coronary heart disease who are prone to death after PCI or poor prognosis such as MACE and MACCE events, and to follow up high-risk patients closely. Further studies are needed to confirm the findings of GAR in relation to adverse events after PCI in patients with coronary heart disease.

## Data Availability

The datasets used and/or analyzed during the current study available from the corresponding author on reasonable request.
